# Managing obesity in primary care practice: a narrative review

**DOI:** 10.1111/nyas.12004

**Published:** 2013-01-16

**Authors:** Raymond Carvajal, Thomas A Wadden, Adam G Tsai, Katherine Peck, Caroline H Moran

**Affiliations:** 1 Department of Psychiatry, Center for Weight and Eating Disorders, Perelman School of Medicine, University of PennsylvaniaPhiladelphia, Pennsylvania; 2 Department of Medicine, Division of General Internal Medicine, Anschutz Health and Wellness Center, University of Colorado DenverDenver, Colorado

**Keywords:** obesity, weight loss, primary care

## Abstract

This narrative review examines randomized controlled trials of the management of obesity in primary care practice, in light of the Centers for Medicare and Medicaid Services’ decision to support intensive behavioral weight loss counseling provided by physicians and related health professionals. Mean weight losses of 0.1–2.3 kg were observed with brief (10- to 15-min) behavioral counseling delivered by primary care providers (PCPs) at monthly to quarterly visits. Losses increased to 1.7–7.5 kg when brief PCP counseling was combined with weight loss medication. Collaborative treatment, in which medical assistants delivered brief monthly behavioral counseling in conjunction with PCPs, produced losses of 1.6–4.6 kg in periods up to two years. Remotely delivered, intensive (>monthly contact) behavioral counseling, as offered by telephone, yielded losses of 0.4–5.1 kg over the same period. Further study is needed of the frequency and duration of visits required to produce clinically meaningful weight loss (>5%) in primary care patients. In addition, trials are needed that examine the cost-effectiveness of PCP-delivered counseling, compared with that potentially provided by registered dietitians or well-studied commercial programs.

## Introduction

The U.S. Preventive Services Task Force (USPSTF) has recommended that clinicians screen all adults for obesity and offer intensive multicomponent behavioral interventions to affected individuals, either by providing such treatment themselves or referring patients to appropriate interventions.^1^ U.S. adults certainly warrant such attention, given that 34% are obese, defined by a body mass index (BMI) of 30 kg/m^2^ or more, and another 32% are overweight (i.e., BMI of 25.0–29.9 kg/m^2^).^2^ In accordance with the task force's recommendations, the Centers for Medicare and Medicaid Services (CMS) recently approved the provision of intensive behavioral counseling to obese seniors in primary care practice when delivered by physicians, nurse practitioners (NPs), or physician assistants (PAs) from the practice.^3^ CMS recommended that these practitioners provide brief (15 min) weekly counseling sessions for the first month, followed by every-other-week visits for an additional five months. Patients who lose ≥3 kg in the first six months (in these 14 sessions) are eligible for six additional monthly visits.

CMS's decision to provide behavioral counseling for obesity is laudable, given the demonstrated benefits of this approach in reducing weight and cardiovascular disease (CVD) risk factors, particularly type 2 diabetes.^4^–^7^ The stipulation, however, that behavioral intervention be provided by physicians, NPs, and PAs (from the practices) is surprising. We are not aware of any randomized controlled trials (RCTs) that have assessed the efficacy of brief counseling visits, as delivered by these providers, on the schedule mandated by CMS. In short, CMS's treatment model is not supported by an adequate evidence base.

The present narrative review updates an earlier examination of the treatment of obesity by primary care practitioners (PCPs) who delivered lifestyle counseling to overweight/obese patients in their practices.^8^ In addition, the review examines different potential models for managing obesity in primary care, including the options of incorporating auxiliary health professionals on the treatment team or referring patients to providers or programs outside of the practice, as suggested by the Task Force.^1^

## Behavioral interventions for obesity

Behavioral treatment for obesity—consisting of a combination of diet, physical activity, and behavior therapy—is considered the cornerstone of weight management for overweight/obese adults.^5^,^6^ This approach uses behavioral strategies, such as goal setting and record keeping, to help individuals reduce their calorie intake by approximately 500–1,000 kcal/day, principally by reducing their portion sizes, snacking, and consumption of high-fat, high-sugar foods.^4^,^7^,^9^ Caloric restriction is combined with recommendations to exercise (e.g., brisk walking) for at least 30 min/day most days of the week (i.e., 180 min/week).^10^ In academic medical centers, behavioral treatment typically is delivered in weekly group or individual sessions that are led by registered dietitians, psychologists, exercise specialists, and other counseling professionals. Weekly group lifestyle intervention of 16–26 weeks induces a mean weight loss of approximately 7–10% of initial weight during this time.^4^,^11^,^12^

A recent systematic review by the task force^13^ revealed the importance of providing high-intensity behavioral interventions (defined in a prior Task Force report as “more than once a month, face-to-face contact during the first three months”).^14^ Interventions that provided 12–26 treatment sessions in the first year generally induced weight losses of 4–7 kg, whereas those that offered fewer than 12 sessions yielded losses of only 1.5–4 kg.^13^ This latter finding extends the USPTF's earlier conclusion that there was sufficient evidence to recommend the prescription of high-intensity counseling, but not moderate-intensity (defined as monthly contact) or low-intensity (i.e., < monthly) behavioral counseling.^14^

The task force's conclusions were based on its review of what it described as “primary care-relevant treatments for obesity.”^13^ However, the largest weight losses reported in the review were observed in RCTs conducted in academic medical centers, which employed experienced lifestyle interventionists (i.e., registered dietitians, psychologists, and exercise specialists) who provided weekly group or individual treatment for the first three months or more.^7^,^15^–^17^ Group treatment sessions generally lasted 60–90 min (with individual sessions of 30 min), and some trials included supervised exercise training.^17^ A pressing question is whether these interventions, which may be “relevant” to primary care, could actually be implemented in busy primary care practices using the practice's available physicians and NPs. These professionals typically have little formal training in behavioral weight management.^18^ Moreover, 15-min counseling sessions, as proposed by CMS, provide minimal time to review patients’ eating and activity records and to identify solutions to problems identified.

## Models for providing behavioral weight management in primary care

**Figure 1:**
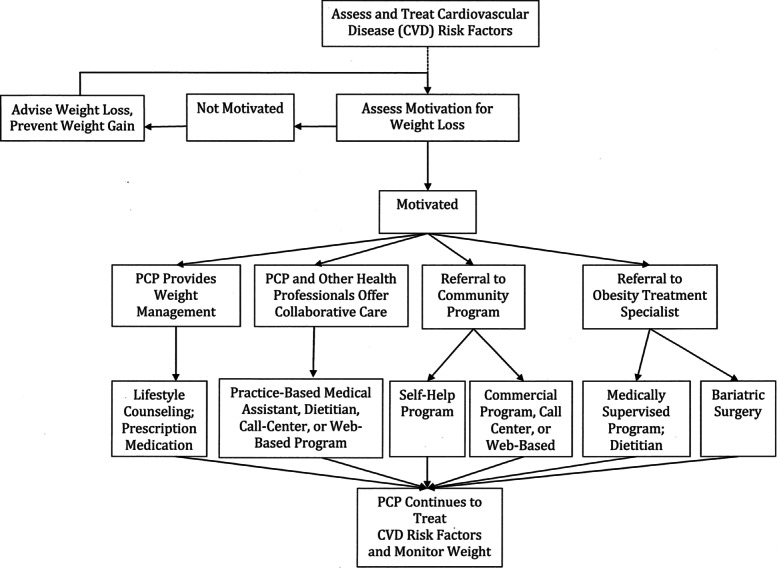
An algorithm for identifying an appropriate weight loss option. After treating cardiovascular disease (CVD) risk factors and assessing patients’ activation for weight loss, primary care providers (PCPs) may elect to offer behavioral counseling themselves (with or without pharmacotherapy) or to provide collaborative care with other health professionals. Alternatively, PCPs may refer patients to community programs (e.g., Weight Watchers) or to obesity treatment specialists (e.g., medically supervised programs, bariatric surgery).

Tsai and Wadden^8^ have proposed several treatment models for engaging PCPs in the management of obesity and for providing behavioral weight management to appropriate patients (see Fig. 1). PCPs play a critical role in screening adults for obesity and in providing appropriate medical management for weight-related CVD risk factors (e.g., hypertension, type 2 diabetes) and other conditions (osteoarthritis).^9^ PCPs also are well prepared to educate patients about the contribution of excess weight to health complications, as well as to inform them of the significant health benefits of a 5–10% reduction in initial weight.^5^,^9^ Health professionals also can assess obese patients’ motivation for weight reduction and, with interested patients, develop a weight loss plan. With patients who do not wish to lose weight, PCPs can use motivational interviewing to clarify barriers to treatment and then discuss the need to prevent further weight gain.^19^

PCPs have multiple options for offering behavioral weight loss counseling. They may themselves provide lifestyle counseling to patients during routine office visits. As shown on the left hand side of Figure 1, lifestyle counseling may be provided alone or in combination with weight loss medication, given the increased loss that results from combining these two approaches.^20^ PCPs who cannot provide behavioral counseling themselves, because of limited time or competing practice demands, could have auxiliary health professionals in their practice offer such counseling. Nurses, medical assistants, and other personnel could be trained as “lifestyle coaches,”^21^ or the practice could hire a registered dietitian or behavioral psychologist to provide counseling. Tsai and Wadden^8^ have referred to this as “collaborative obesity care.” In all cases, patients would receive behavioral weight management within the primary care practice, which has the potential advantage of capturing individuals at the point of treatment and fully integrating weight management with patients’ other health care.

The provision of behavioral counseling using either of these models may be impractical in many primary care practices because of the increased volume of patient visits (resulting from high-frequency counseling), lack of physical space, or costs of hiring additional staff. Some PCPs may be able to refer patients to programs or professionals who provide counseling as part of an integrated health system. This may include the use of call centers that provide counseling remotely. Other options include face-to-face self-help or commercial programs in the community that have been empirically validated (e.g., Weight Watchers). Alternatively, PCPs could refer patients to obesity-treatment specialists in the community (e.g., registered dietitians, physicians, bariatric surgeons). With all of these options, patients will benefit from the PCP playing an active role in monitoring changes in their weight and health, congratulating them on their successes, and reminding them of the need for long-term behavior change.

## Evidence supporting the different models of behavioral intervention in primary care

The next sections of this review examine studies relevant to the different models of behavioral intervention described earlier. For studies of PCP counseling and collaborative obesity treatment, we identified trials that were conducted in primary care practice and incorporated health care providers from the practice. Several studies were included that attempted to simulate primary care practice by using PCPs inexperienced in weight management as lifestyle interventionists in randomized trials. Thus, this review differs from the recent Task Force^13^ analysis which examined primary care relevant studies, many of which included highly trained lifestyle interventionists and were not conducted in primary care settings. We thought that limiting studies in this manner would provide the most appropriate estimates of the results that could be expected from the CMS proposal to have primary care providers deliver intensive behavioral counseling to obese patients in their home practices. (The results of high-intensity behavioral interventions, as delivered in RCTs in academic medical centers, have been reported in numerous other reviews.^4^,^11^,^12^)

## Brief behavioral weight loss counseling provided by PCPs

Four randomized trials^22^–^26^ assessed the effects of physician-delivered weight loss counseling in primary care practice. Martin *et al.*^22^ randomly assigned low-income African-American women, with a mean age of 41.7 years and BMI of 38.8 kg/m^2^, to either usual care, consisting of as-needed medical treatment, or a six-month weight loss intervention, consisting of brief monthly PCP counseling sessions. Counseling visits lasted approximately 15 min and included personalized recommendations for changing diet and physical activity. At six months, patients who received PCP counseling lost a mean of 1.4 kg, compared with a gain of 0.3 kg for usual care (*P* = 0.01). However, there were no significant differences between groups at the 18-month follow-up,^23^ as shown in Table 1.

**Table 1:** Studies of brief primary care provider (PCP) counseling, provided alone or with meal replacements or pharmacotherapy

Study	*N*	Interventions	Number of treatment visits	Month of postrandomization follow-up	Weight change at month 6, kg	Weight change at follow-up, kg	≥5% loss of initial weight at follow-up, % of subjects	Attrition at follow-up, %*
Brief PCP counseling								
Christian *et al.*^24^	310	1. Quarterly PCP visits	4	12	—	+0.6 ± 0.4^a^	11^a^	15
		2. Quarterly PCP visits+ PCP counseling	4	12	—	−0.1 ± 0.4^a^	21^b^	9
Cohen *et al.*^26^	30	1. Usual care	5.2	12	+0.6 ± 0.6^a^	+1.3 ± 0.8^a^	–	Not stated
		2. Usual care + PCP counseling	9.7	12	−1.8 ± 0.9^b^	−0.9 ± 1.0^a^	–	
Martin *et al.*^22^,^23^	144	1. Usual care	0	18	+0.3 ± 0.4^a^	+0.1 ± 0.5^a^	12^a^	23
		2. Usual care + PCP counseling	6	18	−1.4 ± 0.5^b^	−0.5 ± 0.4^a^	7^a^	44
Ockene *et al.*^25^**	1,162	1. Usual care	3.4	12	—	0.0^a^	–	42
		2. PCP training	3.1	12	—	−1.0^ab^	–	42
		3. PCP training + office support	3.6	12	—	−2.3^b^	–	37
Brief PCP counseling + meal replacements								
Ashley *et al.*^27^	113	1. RD counseling	26	12	—	−3.4 ± 1.1^a^	–	38
		2. RD counseling + meal replacements	26	12	—	−7.7 ± 1.5^b^	–	32
		3. PCP/RN counseling + meal replacements	26	12	—	−3.5 ± 1.1^a^	–	34
Brief PCP counseling + pharmacotherapy								
Hauptman *et al.*^31^	635	1. PCP guidance + placebo	10	24	−4.7 ± 0.6^a^	−1.7 ± 0.6^a^	24.1^a^	57
		2. PCP guidance + orlistat, 60 mg TID	10	24	−6.9 ± 0.6^b^	−4.5 ± 0.6^b^	33.8^b^	44
		3. PCP guidance + orlistat, 120 mg TID	10	24	−8.0 ± 0.6^b^	−5.0 ± 0.7^b^	34.3^b^	44
Poston *et al.*^32^	250	1. RD/RN counseling	13	12	+0.6 ± 0.3^a^	+1.7 ± 0.5^a^	9.4	67
		2. Orlistat, 120 mg TID	13	12	−2.3 ± 0.6^b^	−1.7 ± 0.8^b^	24.1	35
		3. RD/RN counseling + orlistat, 120 mg TID	13	12	−2.9 ± 0.5^b^	−1.7 ± 0.7^b^	26.8	34
Wadden *et al.*^34^†	106	1. Sibutramine, 10–15 mg daily	8	12	—	−5.0 ± 1.0^a^	42	18
		2. Sibutramine, 10–15 mg daily + PCP counseling	8	12	—	−7.5 ± 1.1^a^	56	19

Note: Values shown for weight change are mean ± SEM. For each study, under “weight change” (at month 6 and at follow-up) and “≥5% loss of initial weight at follow-up,” values labeled with different letters (a, b) are significantly different from each other at *P* < 0.05.

* Attrition is defined as the percentage of participants who did not contribute an in-person weight at the end of the study. An intention-to-treat analysis was used in most studies, except for three that used a completers’ analysis.^25^–^27^

** This study did not report the standard deviations or standard errors of weight loss.

† This study included two additional groups, both of which included intensive group lifestyle modification. The results of these groups are not displayed here.

RD, registered dietitian; RN, registered nurse; TID, three times per day.

Christian *et al.*^24^ examined PCP counseling in patients with type 2 diabetes, with a mean age of 53.2 years and BMI of 35.1 kg/m^2^. Patients were randomly assigned to either a control group, consisting of quarterly PCP visits and printed health education materials, or a lifestyle intervention that included brief PCP-delivered motivational interviewing during quarterly visits. Patients in the intervention group completed computer-based assessments of their motivation for lifestyle change, which PCPs used to guide their counseling recommendations. At 12 months, the intervention group lost 0.1 kg, compared with a gain of 0.6 kg in controls (*P* = 0.23).

Ockene *et al.*^25^ evaluated the effects of brief PCP counseling in patients with hyperlipidemia, with a mean age of 49.3 years and BMI of 28.7 kg/m^2^. Forty-five PCPs were randomly assigned to provide one of three interventions: usual care, physician-delivered nutrition counseling, or physician-delivered nutrition counseling, plus an office-support program. The nutrition intervention was based on a brief patient-centered counseling model, with sessions of 8–10 minutes. The office support program assisted PCPs in implementing the counseling protocol by providing in-office prompts and counseling algorithms. Patients in the three groups were seen an average of 3.4 times over one year. Those who received the combination of physician-delivered counseling and office support lost significantly more weight (2.3 kg) at one year than those in usual care (0.0 kg; *P* < 0.001). There were no significant differences between the group that received physician-delivered counseling alone (–1.0 kg) and the two other treatment arms.

In a similar study, Cohen *et al.*^26^ assessed the effectiveness of PCP counseling in patients with hypertension, with a mean age of 59.5 years and BMI of 34.1 kg/m^2^. Eighteen resident physicians were randomly assigned to usual care or to nutrition counseling training. Those in the latter group were instructed in standard weight loss methods (e.g., calorie control, healthy food alternatives), and their patients were offered monthly counseling visits. Patients in the two groups had an average of 5.2 and 9.7 visits, respectively, over one year. At month six, patients of physicians who received nutrition counseling training lost 1.8 kg, compared with a gain of 0.6 kg for usual care (*P* = 0.04). However, as shown in Table 1, there were no significant differences between groups at one year.

### Summary

Collectively, these four studies^22^–^26^ suggest that low- to moderate-intensity, brief lifestyle counseling, provided by PCPs, is unlikely to produce clinically significant weight loss (≥5% of initial weight) in overweight/obese patients (although losses of 2–4.9% may have some clinical benefit^6^). Weight losses in the intervention arms ranged from only 0.1 to 2.3 kg, losses that generally are not associated with significant health improvements.^6^ (In most cases, a loss of 5 kg corresponds to a loss of 5% of initial weight.) The low frequency of treatment contact is likely responsible for these modest outcomes, although the brief duration of treatment visits (i.e., 10–15 min) also may be a factor. Increasing the frequency of PCP visits to at least twice monthly could be expected to increase weight loss, as suggested by the Task Force's review.^13^ This possibility is suggested by results of a study by Ashley *et al.*^27^ in which PCPs delivered brief behavioral counseling to community volunteers recruited from advertisements (i.e., not primary care patients), as shown in Table 1. Participants, who had a mean age of 40.4 years and BMI of 30.0 kg/m^2^, were assigned to (1) group behavioral counseling, delivered by registered dietitians in 26 one-hour sessions; (2) the same intervention combined with meal replacements, which have been shown to increase weight loss compared with the use of conventional reducing diets;^28^ or (3) PCP counseling delivered in 26 brief (10–15 min) biweekly sessions that included the use of meal replacements. All participants received the LEARN manual,^29^ which was the basis of counseling sessions. At one year, patients who received group treatment, with or without meal replacements, lost 7.7 kg and 3.4 kg, respectively, whereas those treated by PCPs lost 3.5 kg (the first condition was superior to the two others, *P* = 0.03; these results are for a completers’ analysis). Further study is needed to determine whether the 3.5 kg loss achieved by PCPs was attributable to the high frequency of visits (i.e., 26 in one year), the use of meal replacements, or the combination of the two factors.

## Brief PCP counseling plus pharmacotherapy

Trials in academic medical centers have shown that adding weight loss medication to lifestyle counseling increases mean weight loss.^20^,^30^ Three RCTs^31^,^32^,^34^ examined the effectiveness of lifestyle counseling plus pharmacotherapy, provided by PCPs as part of interventions that modeled brief office visits in primary care. Hauptman *et al.*^31^ studied the effectiveness of orlistat (a gastric and pancreatic lipase inhibitor) in primary care patients, with a mean age of 42.5 years and BMI of 36 kg/m^2^, who were randomly assigned to placebo, 60 mg of orlistat TID, or 120 mg of orlistat TID. All patients were prescribed a reduced-calorie diet during year one and a weight-maintenance diet during year two. They also received brief dietary guidance from their PCPs, along with educational videotapes and printed materials. As shown in Table 1, weight losses at month 24 were 1.7, 4.5, and 5.0 kg for the three groups, respectively (*P* = 0.001 for both orlistat groups compared to placebo).

Poston *et al.*^32^ also tested orlistat in a primary care simulated study of patients with an average age of 41.0 years and BMI of 36.1 kg/m^2^. Participants were randomly assigned to brief weight loss counseling alone, consisting of 15- to 20-min monthly sessions with a nurse or registered dietitian, orlistat alone (120 mg TID), or the combination of brief weight loss counseling and orlistat. Patients in the counseling arms received the LEARN manual.^33^ At 12 months, the groups that received orlistat alone or combined with brief weight loss counseling both lost a mean of 1.7 kg, compared with a gain of 1.7 kg for lifestyle counseling alone (*P* < 0.001 for both orlistat groups compared to counseling alone). There were no significant differences between the two medication groups.

In a similar study, Wadden *et al.*^34^ assessed the effects of sibutramine, a serotonin–norepinephrine reuptake inhibitor, which was removed from the market in 2010 because of concerns that it increased the risk of cardiovascular events.^35^ Patients had a mean age of 43.6 years and BMI of 37.9 kg/m^2^ and were randomly assigned to sibutramine (10–15 mg daily), accompanied by eight brief PCP visits over 12 months to monitor blood pressure and pulse, or sibutramine plus brief PCP lifestyle counseling, provided during the eight brief visits. Patients in the latter group completed homework assignments from the LEARN manual,^36^ including daily food and activity records. Patients who received sibutramine plus PCP counseling lost significantly more weight at week 18 than did those who received sibutramine alone (8.4 kg vs. 6.2 kg, *P* = 0.05). At month 12, however, differences between groups were no longer significant.

### Summary

These three studies^31^,^32^,^34^ suggest that the addition of pharmacotherapy to brief PCP-delivered lifestyle counseling will increase the likelihood that patients achieve clinically meaningful weight loss (≥5% of initial weight), as compared with brief counseling alone. This conclusion, however, is tempered by the fact that sibutramine was removed from the market,^35^ and there have not been trials, in primary care settings, of two newly approved weight loss medications.^37^,^38^ Studies are needed to determine whether lorcaserin,^37^ as well as the combination of phentermine/topiramate,^38^ significantly improve weight loss when added to brief PCP lifestyle counseling, compared with counseling alone. Cost-effectiveness analyses will be required in these trials, given the expense of the medications.

## Collaborative obesity treatment within primary care practice

Four recent studies^21^,^40^,^42^,^43^ evaluated the effectiveness of collaborative obesity treatment, in which auxiliary health professionals were trained to provide weight loss counseling in conjunction with a PCP who addressed patients’ routine medical care. Tsai *et al.*^21^ developed a model in which medical assistants served as lifestyle interventionists (coaches). Patients, with a mean age of 49.5 years and BMI of 36.5 kg/m^2^, were randomly assigned to a control group, consisting of quarterly PCP visits and printed weight loss materials, or to brief counseling, which included quarterly PCP visits, along with eight brief (15–20 min) counseling sessions with a trained medical assistant. The lifestyle intervention was adapted from the Diabetes Prevention Program (DPP).^39^ At six months, patients in the brief counseling and control groups lost 4.4 kg and 0.9 kg, respectively (*P* < 0.001). In addition, 48% of patients in the former group lost 5% or more of their weight, compared to 0% in the control group (*P* = 0.0001). However, as shown in Table 2, there were no significant differences between groups at a one-year follow-up (as a result of weight regain following treatment termination at month six).

**Table 2:** Studies of collaborative obesity care that included auxiliary health professionals in the site's primary care practice

Study	*N*	Interventions	Number of treatment visits	Months of postrandomization follow-up	Weight change at month 6, kg	Weight change at follow-up, kg	≥5% loss of initial weight at follow-up, % of subjects	Attrition at follow-up, %*
PCP + auxiliary health professionals								
Kumanyika *et al.*^42^	261	1. Brief PCP counseling	4	12	—	−0.6 ± 0.4^a^	10.2^a^	28
		2. Brief PCP counseling + MA counseling	16	12	—	−1.6 ± 0.5^a^	22.5^b^	28
ter Bogt *et al.*^43^	457	1. Usual care	1	12	—	−0.9 ± 0.3^a^**	—	7
		2. NP counseling	5	12	—	−1.9 ± 0.3^b^**	—	11
Tsai *et al.*^21^	50	1. Quarterly PCP visits	4	12	−0.9 ± 0.6^a^	−1.1 ± 0.8^a^	12^a^	4
		2. Quarterly PCP visits + MA counseling	12	12	−4.4 ± 0.6^b^	−2.3 ± 0.9^a^	18^a^	8
Wadden *et al.*^40^	390	1. Usual care	8	24	−2.0 ± 0.5^a^	−1.7 ± 0.7^a^	21.5^a^	15
		2. Brief lifestyle counseling (quarterly PCP visits + MA counseling)	33	24	−3.5 ± 0.5^b^	−2.9 ± 0.7^ab^	26.0^ab^	15
		3. Enhanced brief lifestyle counseling (quarterly PCP visits + MA counseling + meal replacements/ medication)	33	24	−6.6 ± 0.5^c^	−4.6 ± 0.7^b^	34.9^b^	12
PCP + multidisciplinary team								
Ryan *et al.*^44^	390	1. Usual care	2	24	—	0.0 ± 0.4^a^**	9^a^	55
		2. Counseling† + meal replacements + medication	46	24	—	−8.3 ± 0.8^b^ **	31^b^	49

Note: Values shown for weight change are mean ± SEM. For each study, under “weight change” (at month 6 and at follow-up) and “≥5% loss of initial weight at follow-up,” values labeled with different letters (a, b, c) are significantly different from each other at *P* < 0.05.

* Attrition is defined as the percentage of participants who did not contribute an in-person weight at the end of the study. An intention-to-treat analysis was used in these studies.

** Weight losses represent percentage weight change.

† In this study, lifestyle counseling was provided by a registered dietitian, social worker, professional counselor, or marriage and family therapist who was not necessarily from the primary care practice site.

PCP, primary care provider; MA, medical assistant; NP, nurse practitioner.

**Figure 2:**
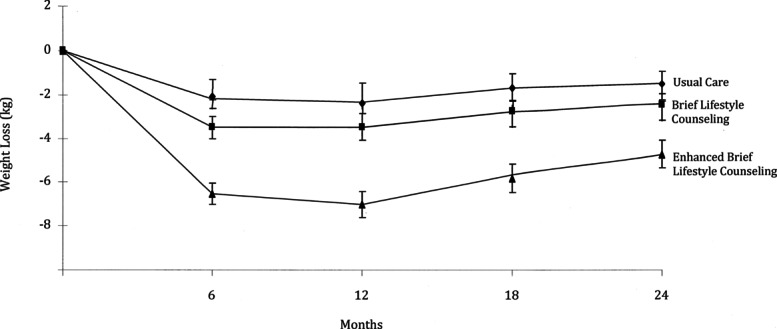
Change in weight over 24 months in three randomized groups. At month 24, enhanced brief lifestyle counseling resulted in significantly greater weight loss than did usual care (*P* = 0.003), with no other significant differences between groups. Reprinted from Ref. 40.

Wadden *et al.*^40^ expanded on the previous study by assessing the effects of brief monthly lifestyle counseling, delivered by medical assistants, over a two-year period. This investigation was one of three independent, yet coordinated trials (collectively known as the Practice-based Opportunities for Weight Reduction (POWER) trials) that were funded by the NHLBI to test the effectiveness of behavioral weight loss counseling in primary care settings.^41^ Participants had a mean age of 51.5 years and BMI of 38.5 kg/m^2^ and were randomly assigned to (1) usual care, consisting of quarterly PCP visits and printed weight loss materials, (2) brief lifestyle counseling, consisting of quarterly PCP visits combined with brief monthly counseling sessions with a trained medical assistant, or (3) enhanced brief lifestyle counseling, which was the same as the second arm, but allowed patients to use meal replacements or weight loss medication (orlistat or sibutramine). The medical assistants followed abbreviated lessons adapted from the DPP,^39^ with visits of 10–15 minutes. Weight losses in the three groups at month six were 2.0, 3.5, and 6.6 kg, respectively, with all three groups differing significantly (*P* < 0.05) from each other. As shown in Figure 2, weight losses at 24 months were 1.7, 2.9, and 4.6 kg, respectively. Weight decreased by ≥ 5% in 21.5, 26.0, and 34.9% of patients in the three groups, respectively. Enhanced brief lifestyle counseling was superior to usual care on both measures of success (*P* = 0.003 and *P* = 0.02, respectively). A secondary analysis that removed participants treated by sibutramine, and examined those treated principally by meal replacements, revealed a loss of 4.1 kg in the enhanced counseling group (which differed significantly from usual care; *P* < 0.05).

Kumanyika *et al.*^42^ compared the results of low- versus moderate-intensity behavioral counseling, as defined by the Task Force (i.e., < monthly contact vs. once-a-month sessions).^14^ Patients had a mean age of 47.2 years and BMI of 37.2 kg/m^2^ and were randomly assigned to either low-intensity care, consisting of brief PCP counseling every four months, or to moderate-intensity care, which included brief PCP counseling every four months, plus brief (15–20 min) monthly lifestyle counseling visits with a trained auxiliary provider (e.g., medical assistant). At one year, patients in the two groups lost 0.6 kg and 1.6 kg, respectively (*P* = 0.15).

A study by terBogt *et al.*^43^ compared weight losses in patients, with an average age of 56.1 years and BMI of 29.6 kg/m^2^, assigned to either usual care or lifestyle counseling with a NP. The counseling arm consisted of four face-to-face visits and one telephone session with the NP, who followed a set of computerized treatment guidelines. At month 12, participants in the intervention and usual care groups lost 1.9% and 0.9% of initial weight, respectively (*P* < 0.05).

### Summary

The results of these four studies^21^,^40^,^42^,^43^ suggest that collaborative obesity treatment that incorporates auxiliary health providers as lifestyle coaches is modestly more effective than PCP counseling alone in treating obesity in primary care settings. The greater weight loss is probably attributable to the greater frequency of visits offered by auxiliary health professionals (typically monthly) as compared with PCPs (typically quarterly). However, weight losses remained modest with collaborative treatment, ranging from 1.6 to 4.6 kg in the intervention arms. The most favorable results were observed with patients who received monthly lifestyle counseling from medical assistants, combined with meal replacements or weight loss medication.^40^

The benefit of increasing the number of treatment modalities (e.g., meal replacements, pharmacotherapy), as well as treatment intensity (i.e., high-frequency visits), was demonstrated by Ryan *et al.*^44^ in a pragmatic trial that created obesity specialty clinics for extremely obese patients covered by a common insurance plan. Patients had a mean age of 47.2 years and median BMI of 46.1 kg/m^2^. They were self-referred to one of seven regional specialty clinics, established at existing primary care practices, and were randomly assigned to either usual care, which included instructions for an internet weight loss website, or an intensive medical intervention, which initially consisted of a three-month low-calorie, liquid diet (890 kcal/day), followed for the next four months by 10 sessions of group behavior modification, combined with weight loss medication (e.g., sibutramine, orlistat, or diethlypropion). From months 8–24, participants were offered monthly group lifestyle modification, weight loss medication, and one meal replacement per day. Lifestyle counselors were drawn, when possible, from primary care staff and included a registered dietitian, social worker, professional counselor, and marriage and family therapist. As shown in Table 2, patients in the intervention group lost 8.3% of initial weight at month 24, compared with 0% for the control group (*P* < 0.001), as determined by a last-observation-carried-forward analysis. (Data also were analyzed using a baseline-carried-forward analysis, in view of two-year attrition of approximately 50%, and revealed mean losses of 4.9% and 0.2%, respectively.) These results indicate that PCPs can be trained to provide treatments that will induce clinically meaningful weight loss in their obese patients. Additional study is required to determine whether such treatment is cost-effective, compared with other interventions that may be available in the community.

## Collaborative obesity treatment supported by remotely delivered counseling

Behavioral weight loss counseling is increasingly being delivered by telephone,^45^–^47^ smart phones,^48^ and the Internet.^49^,^50^ Remotely delivered counseling induces somewhat smaller weight losses than face-to-face interventions^50^ but appears to be less expensive and more convenient for patients (i.e., no travel costs) and capable of reaching more individuals (particularly those in rural areas). Remote delivery, using call centers and similar methods, also could appeal to primary care practices by reducing patient volume at the site, while also providing external expertise (i.e., weight loss counseling) that primary care staff may not possess. Technological advances also could allow call center staff to communicate patients’ progress to primary care staff, if the two parties were part of an integrated health system.

**Figure 3:**
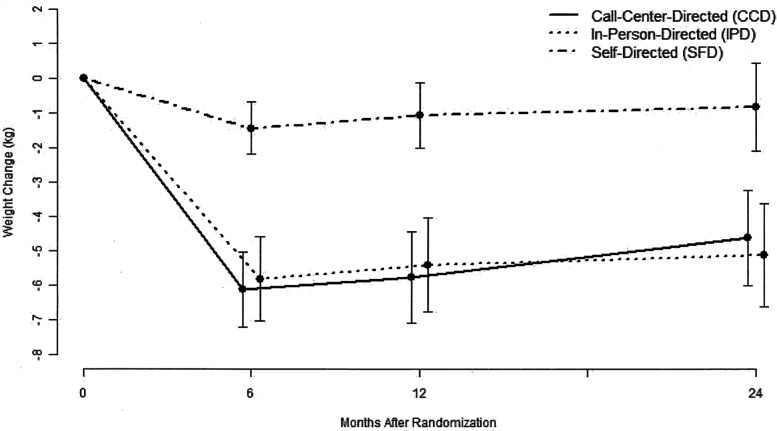
Mean weight change according to randomized group (Call-Center–Directed = remote support only; In-Person–Directed = in-person support; Self-Directed = control). At month 24, both intervention groups lost significantly more weight than the control group (*P* < 0.001), with no significant difference between the intervention groups. Reprinted from Ref. 47.

Five studies^47^,^51^–^54^ have examined the use of remotely delivered counseling with patients in primary care practices (see Table 3). Appel *et al.*^47^ examined the effectiveness of a behavioral weight loss intervention delivered remotely or in-person, in both cases by interventionists not affiliated with the primary care practices. (This investigation was one of the POWER trials, described previously.) Patients, with a mean age of 54.0 years and BMI of 36.6 kg/m^2^, were randomly assigned to (1) a control group, in which weight loss was self-directed; (2) remote support only, consisting of a behavioral weight loss intervention delivered by phone (i.e., 12 initial weeks of 20-min calls, followed by similar monthly calls), e-mail, and Internet; or (3) in-person support, which included the same components provided in the second arm, with the addition of individual and group counseling sessions (participants in the two intervention groups were offered a total of 33 and 57 treatment contacts, respectively). Trained health coaches delivered both interventions. At month 24, weight losses in the three groups were 0.8, 4.6, and 5.1 kg, respectively (see Fig. 3). Weight decreased by 5% or more in 18.8, 38.2, and 41.4% of patients in the three groups, respectively. Both intervention groups were superior to usual care on both measures of success (*P* < 0.001).

**Table 3:** Studies of collaborative obesity care supported by remotely delivered counseling

Study	*N*	Interventions	Number of treatment visits	Months of postrandomization follow-up	Weight change at month 6, kg	Weight change at follow-up, kg	≥5% loss of initial weight at follow-up, % of subjects	Attrition at follow-up, %*
Appel *et al.*^47^	415	1. Control (self-directed)	2	24	−1.4 ± 0.4^a^	−0.8 ± 0.6^a^	18.8^a^	7
		2. Remote support only (telephone + electronic-based counseling)	33	24	−6.1 ± 0.5^b^	−4.6 ± 0.7^b^	38.2^b^	5
		3. In-person support (telephone + electronic-based + in-person counseling)	57	24	−5.8 ± 0.6^b^	−5.1 ± 0.8^b^	41.4^b^	4
Bennett *et al.*^54^	101	1. Usual care	0	3	—	0.3 ± 0.3^a^	0	16
		2. Web-based + brief RD counseling	4	3	—	−2.3 ± 0.5^b^	25.6	16
Bennett *et al.*^53^	365	1. Usual care	0	24	−0.1 ± 0.4^a^	−0.5 ± 0.4^a^	19.5	10
		2. Telephone + electronic-based + group counseling	30	24	−1.3 ± 0.4^b^	−1.5 ± 0.4^b^	20.0	18
Ely *et al.*^52^	101	1. Patient education	0	6	—	−1.0 ± 0.9^a^	—	52
		2. Patient education + telephone counseling	8	6	—	−4.3 ± 0.8^b^	—	48
Logue *et al.*^51^	665	1. Brief RD counseling	4	24	—	−0.2 ± 0.4^a^	—	31
		2. Brief RD counseling + telephone counseling	28	24	—	−0.4 ± 0.4^a^	—	38

Note: Values shown for weight change are mean ± SEM. For each study, under “weight change” (at month 6 and at follow-up) and “≥5% loss of initial weight at follow-up,” values labeled with different letters (a, b) are significantly different from each other at *P* < 0.05.

* Attrition is defined as the percentage of participants who did not contribute an in-person weight at the end of the study. An intention-to-treat analysis was used in most studies, except for one that used a completers’ analysis.^52^

RD, registered dietitian.

Logue *et al.*^51^ conducted a two-year study of a telephone-delivered weight loss intervention in primary care practice. Overweight and obese patients, ranging in age from 40 to 60 years, were randomly assigned to either augmented usual care, consisting of 10-min, semiannual counseling sessions with a dietitian, or transtheoretical model-chronic disease care, which included the same components as the first arm, accompanied by 15-min monthly telephone calls with a weight loss advisor trained in stages-of-change interventions. Patients in the latter group also were mailed stage-specific, behavioral weight loss materials. After two years, weight losses in the two groups were 0.2 kg and 0.4 kg, respectively (*P* = 0.5).

Ely *et al.*^52^ examined the treatment of obesity in rural primary care settings. Patients, with an average age of 49.5 years and BMI of 36 kg/m^2^, were randomly assigned to either usual care, which included educational weight loss materials, or a chronic care intervention consisting of eight telephone-delivered motivational interviewing sessions with a masters-level counselor. PCPs also provided personalized weight loss recommendations to patients in the latter group. Weight losses at month six were 1.0 kg and 4.3 kg for the usual care and intervention groups, respectively (*P* = 0.01).

In another POWER trial, Bennett *et al*.^53^ tested the effectiveness of a primary care intervention in predominantly low-income patients with hypertension. Patients, with a mean age of 54.5 years and BMI of 37 kg/m^2^, were randomly assigned to either usual care or a behavioral intervention for weight loss and hypertension self-management. The intervention was delivered through a study-specific website and an interactive voice response system, both of which provided patients with tailored feedback. Patients in the intervention group also were offered 12 group support sessions and received 18 telephone-counseling calls from trained community health educators. Weight losses at month 24 were 0.5 kg and 1.5 kg in the usual care and intervention groups, respectively (*P* < 0.05).

Bennett *et al.*^54^also examined the effects of a web-based intervention in primary care patients with hypertension, who had a mean age of 54.4 years and BMI of 34.6 kg/m^2^. Patients were randomly assigned to (1) usual care, which included printed weight loss materials; or (2) a comprehensive weight loss website that promoted behavior change. Patients in the latter group also received four 20-min motivational coaching sessions delivered by a dietitian, two in-person and two by telephone. At week 12, those in the intervention arm lost 2.3 kg, compared with a gain of 0.3 kg for usual care (*P* < 0.05).

### Summary

These five trials^47^,^51^–^54^ suggest that primary care practices can help their patients achieve clinically meaningful weight loss by offering remotely delivered, high-intensity behavioral counseling. Appel *et al.*^47^ obtained the most promising results, in which 12 weekly telephone sessions, followed by monthly calls thereafter, induced a mean loss of 6.1 kg at month six, with maintenance of a 4.6 kg loss at month 24. However, these patients also received an interactive-web based program in which they recorded their weight, food intake, and physical activity. Thus, the efficacy of telephone-based counseling alone cannot be determined in the present study. Ely *et al.*,^52^ however, also obtained an average 4.3 kg weight loss in six months in a program that provided eight telephone counseling sessions during this time. Studies that provided moderate- or low-intensity phone counseling (often combined with other electronic contact) generally produced smaller weight losses, although randomized trials are needed to assess the effects of intervention intensity. Further studies of call-center–delivered behavioral counseling, which include a cost analysis, clearly are warranted.

## Referral options in the community for PCPs

The right-hand side of Figure 1 shows referral options in the community that are available to PCPs and their patients. These include a variety of community-55,56 and commercial-based programs,^57^,^58^ many of which provide face-to-face meetings and increasingly offer telephone- or Internet-supported counseling. Alternatively, PCPs may refer patients to professionals who specialize in obesity management, including registered dietitians, psychologists, exercise specialists, and bariatric physicians or surgeons. The next few sections only briefly summarize these options. Interested readers are referred to more thorough reviews.^4^,^8^,^59^

### Commercial programs

Two studies^57^,^58^ evaluated the effectiveness of a commercial program with patients referred from primary care practices. Jebb *et al.*^57^ compared the weight losses of 772 patients, with a mean age of 47.4 years and BMI of 31.4 kg/m^2^, who were randomly assigned to either usual care (as provided in their primary care practice) or Weight Watchers. Patients assigned to the latter group were given one year free access to an in-person Weight Watchers program in their area. (Weight Watchers offers weekly group support meetings, in combination with a balanced, reduced-calorie diet and instruction to increase physical activity.) At month 12, mean weight losses were 1.8 kg and 4.1 kg in the usual care and Weight Watchers groups, respectively (*P* < 0.0001). Approximately 25% and 45% of participants in the two groups lost 5% of initial weight (*P* < 0.05).

Jolly *et al.*^58^ compared the effectiveness of usual care to several commercial weight loss programs offered in the United Kingdom. A total of 740 overweight/obese adults, referred from primary care practices, were randomly assigned to one of six programs: Weight Watchers; Rosemary Conley; Slimming World; National Health Service (NHS) Size Down; general-practice counseling (in primary care); and pharmacy-based counseling. A seventh randomized arm allowed participants to choose a weight loss program from the six options. Weight losses at 12 weeks ranged from 1.4 kg (general practice counseling) to 4.4 kg (Weight Watchers). Weight Watchers and another commercial program (Rosemary Conley) were superior to the exercise-only comparator group (2.0 kg; *P* = 0.001 for Weight Watchers). Additional data supporting the effectiveness of Weight Watchers in primary care patients were reported from a retrospective, uncontrolled study by Mitchell *et al.*^60^

The efficacy of other commercial programs was examined previously by Tsai and Wadden.^59^ Since that report, both Jenny Craig^61^,^62^ and Nutrisystem^63^ have been evaluated in randomized trials, ranging from three to 24 months. These studies were not conducted in primary care practices, but the generally favorable results would appear relevant to PCPs and their patients. Cost data are needed to help primary care health systems, insurers, and obese individuals identify which programs are both effective and affordable.

### Obesity specialists

PCPs also can refer patients to obesity specialists in the community who can provide more targeted evaluation and additional options for treatment. Specialists include registered dietitians, particularly those who have completed the American Dietetic Association's Weight Management Certification Program, which provides training in the delivery of evidence-based interventions (http://www.cdrnet.org/wtmgmt/CertificateOfTraining.cfm). Bariatric physicians frequently prescribe weight loss medications, such as phentermine and orlistat,^20^ and likely soon will include the newly approved lorcaserin^37^ and the combination of phentermine and topiramate.^38^ To achieve optimal weight loss, medications must be prescribed in combination with behavioral counseling^34^ and must be taken long term to facilitate the maintenance of lost weight.^20^

Medically supervised low- and very-low-calorie diets are another option offered by bariatric physicians.^64^ Numerous studies have reported mean losses of 15–25% of initial weight with these approaches,^65^–^67^ but patients usually regain 35–50% of their weight within two years following treatment.^64^ As a result, few long-term differences in weight loss have been detected in patients prescribed very-low-calorie diets (< 800 kcal/d) versus more moderately restricted diets of conventional food (1,000–1,800 kcal/day).^64^ Medically supervised programs also are likely to cost more than $100 per week, when the costs of food and physician monitoring are included.^59^

PCPs may consider bariatric surgery with patients with extreme obesity (BMI ≥ 40 kg/m^2^) who have not been successful with lifestyle modification and pharmacotherapy.^5^ Patients with a BMI ≥ 35 kg/m^2^ and comorbid medical conditions also may qualify for surgery. In addition, the FDA has approved laparoscopic adjustable gastric banding (LAGB) for patients with a BMI of 30–34.9 kg/m^2^ and type 2 diabetes (although it currently is unclear whether insurance companies will reimburse the cost of surgery for this population). The most commonly used procedures in the United States are Roux-en-Y-gastric bypass (RYGB), LAGB, and sleeve gastrectomy (SG).^68^,^69^ RYGB produces long-term (>2 years) weight loss of 25–30% of initial weight and LAGB of about 15–20%, with SG generally falling in between.^68^–^71^ Weight loss achieved with bariatric surgery is associated with improvements in comorbid conditions, particularly with the remission of type 2 diabetes in patients who undergo RYGB.^68^–^71^ However, bariatric surgery also carries the highest risk of complications, including perioperative mortality, among the weight loss options considered in this review.^72^ Patients also may regain their weight if they fail to adhere to the postoperative diet.^73^

## Conclusions and future directions

The provision of behavioral weight loss counseling by PCPs and auxiliary health providers in primary care practices has met with limited success, in most cases producing mean weight losses of only 1–3 kg in 6–24 months of intervention. These modest weight losses are most likely attributable to infrequent treatment contacts, typically at monthly to quarterly intervals, as well as to the brief duration of visits, usually 10- to 15-min sessions. This low intensity of treatment may be all that can be readily accommodated in busy outpatient practices, in which providers must respond to a variety of acute illnesses that may seem more pressing than obesity.

The low-intensity treatments tested in primary care settings contrast sharply with the weekly group and individual interventions (with 30- to 90-minute sessions) that have been delivered by weight loss specialists (e.g., registered dietitians, psychologists) in academic medical centers and that have produced mean losses of 7–10% of initial weight. It is not fair to compare the results of (underfunded) pilot studies conducted in primary care with findings from costly efficacy trials that often are implemented without sufficient thought concerning whether the intervention can be widely disseminated. However, the efficacy trials do tend to underscore the importance of frequent patient-provider contact (i.e., high-intensity interventions), as revealed by the task force's review.^13^

CMS's mandate that primary care providers offer high-intensity behavioral interventions to their obese patients, thus, seems appropriate, given the importance for weight loss of frequent patient-provider contact. CMS's provision of 14 counseling sessions during the first six months is close to the 16 visits provided in the DPP in the first six months, at which time participants lost a mean of 7 kg. However, the decision to provide 15-min visits with PCPs, rather than the 30-min sessions used in the DPP, is not supported by sufficient evidence demonstrating the efficacy of the shorter visits. Moreover, as revealed by the present review, there is little evidence that physicians, NPs, and PAs can help most obese patients achieve clinically meaningful weight losses (≥5% of initial weight). The study by Ashley *et al.*,^27^ reviewed above, comes closest to meeting CMS's proposed treatment paradigm. For one year, patients had brief (10- to 15-min), every-other-week visits with a physician or nurse who provided behavioral weight loss counseling (following the LEARN Manual), combined with the use of meal replacements (provided free of charge). Participants lost a mean of only 3.5 kg at the end of the year, despite being provided the 26 office visits and the meal replacements, the latter which usually increase weight loss by 30% or more compared with the consumption of a conventional reducing diet.

Physicians, NPs, and PAs in primary care undoubtedly could be instructed in delivering effective behavioral weight loss counseling, in the same manner that auxiliary health professionals were trained to do so in several of the studies reviewed above. However, other professionals, particularly registered dietitians, already possess the knowledge and skills required to provide effective behavioral counseling and can do so at a substantially lower cost than physicians and the other providers currently approved by CMS. Ultimately, physicians and their health care practices must decide whether they can afford to spend their time providing behavioral weight loss counseling, with its demand for weekly and then twice-monthly sessions for the first six months. Practices would have to hire more physicians, NPs, and PAs to provide routine medical care to patients whose former PCPs’ schedules were now filled delivering behavioral weight loss counseling. Hiring registered dietitians and other lifestyle interventionists to counsel obese patients would appear to make more economical sense for primary care practices, integrated health systems, and CMS than deploying physicians, NPs, and PAs in this effort.

Remotely delivered, high-intensity behavioral weight loss counseling was perhaps the most promising approach identified by this review. Appel *et al.*^47^ found that 12 weekly telephone sessions (20-min), followed thereafter by monthly calls, induced a mean loss of 6.1 kg at six months and of 4.6 kg at 24 months. These losses were equivalent to those of participants who were offered a traditional face-to-face intervention that combined group and individual visits. Participants in Appel's study also received an Internet-based program. However, additional trials, conducted outside of primary care practices which used telephone-counseling alone, have found equivalence of this approach with comparable on-site interventions.^45^,^46^,^61^ Remotely delivered lifestyle counseling, whether provided by a primary care practice, or by a call center with which it has contracted, would appear to be a very convenient option for patients. More important, it would support PCPs in their efforts to offer intensive behavioral counseling, as recommended by the Task Force, without overwhelming the practice schedules of already harried providers. Further study is needed to determine whether remotely delivered weight loss counseling is as effective as it appears to be and can delivered, at a minimum, at a lower cost than CMS pays for on-site counseling delivered by PCPs.
